# Production of Salvianolic Acid B in Roots of *Salvia miltiorrhiza* (Danshen) During the Post-Harvest Drying Process

**DOI:** 10.3390/molecules17032388

**Published:** 2012-02-27

**Authors:** Xiao-Bing Li, Wei Wang, Guo-Jun Zhou, Yan Li, Xiao-Mei Xie, Tong-Shui Zhou

**Affiliations:** 1 Research Center of Natural Products, Ministry of Education Key Laboratory for Biodiversity Science and Ecological Engineering, Institute of Biodiversity Science, Fudan University, Shanghai 200433, China; 2 School of Traditional Chinese Pharmacy, Anhui College of Traditional Chinese Medicine, Hefei 230038, China; Email: xiexiaomei9401@sina.com (X.-M.X.)

**Keywords:** Danshen, dynamic variations, post-harvest drying process, salvianolic acid B, tanshinones

## Abstract

Drying is the most common and fundamental procedure in the post-harvest processing which contributes to the quality and valuation of medicinal plants. However, attention to and research work on this aspect is relatively poor. In this paper, we reveal dynamic variations of concentrations of five major bioactive components, namely salvianolic acid B (SaB), dihydrotanshinone I, cryptotanshinone, tanshinone I and tanshinone IIA, in roots of *Salvia miltiorrhiza* (Dashen) during the drying process at different oven temperatures. A minor amount of SaB was found in fresh materials while an noticeable increase in SaB was detected in drying at 50~160 °C. The maximal value occured after 40 min of drying at 130 °C and its variation showed a reverse V-shaped curve. Production of SaB exhibited a significant positive correlation with drying temperatures and a significant negative correlation with sample moistures. The amounts of tanshinones were nearly doubled in the early stage of drying and their variations showed similar changing trends with drying temperatures and sample moistures. The results supported our speculation that postharvest fresh plant materials, especially roots, were still physiologically active organs and would exhibit a series of anti-dehydration mechanisms including production of related secondary metabolites at the early stage of dehydration. Hence, the proper design of drying processes could contribute to promoting rather than reducing the quality of Danshen and other similar medicinal plants.

## 1. Introduction

**Figure 1:**
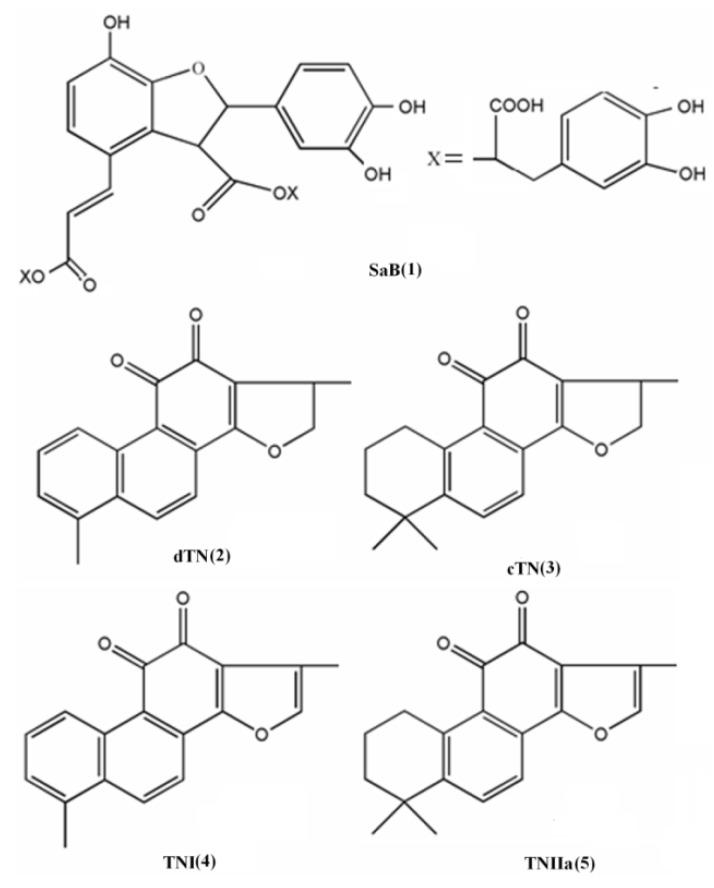
Structures of the five major compounds of Danshen: (1) salvianolic acid B (SaB); (2) dihydrotanshinone I (dTN); (3) cryptotanshinone (cTN); (4) tanshinone I (TNI); (5) tanshinone IIA (TNIIa).

In recent years, various preparations based on Danshen (roots of red sage, *Salvia miltiorrhiza* Bge.) have become popular for patients with cardiovascular diseases both in China and other countries including the United States [1,2,3]. This herb is one of the most important and highly valued Traditional Chinese Medicines (TCMs) and it has been used in the treatment of numerous ailments, including cardiovascular disease, for about 2,000 years. Its pronounced efficacies in improving microcirculation, causing coronary vasodilatation, suppressing the formation of thromboxane, inhibiting platelet adhesion and aggregation, and protecting against myocardial ischemia have attracted worldwide attention [1,2,3]. The bioactivity of this herb is ascribed to an array of components, including hydrophilic caffeic acid derivatives (CaDs) and dozens of lipophilic tanshinones (TNs) [4,5,6,7]. Both of them contribute to its cardioprotective effects, but show significant mechanistic and temporal differences [1,8]. The predominant CaDs is salvianolic acid B (SaB, 1) and the major TNs are dihydrotanshinone I (dTN, 2), cryptotanshinone (cTN, 3), tanshinone I (TNI, 4) and tanshinone IIA (TNIIa, 5) (Figure 1) [7,9].

The commercial materials of this herb are standardized by values of SaB (≥3.0%) and TNIIa (≥0.2%) according to the 2010 Chinese Pharmacopoeia [10]. Up to now, a number of reports on quality evaluations of Danshen and its preparations have been published [7]. These reports demonstrated that values of SaB and TNIIa as well as other ingredients in roots of *S. miltiorrhiza* and its based preparations varied significantly [7,9,11]. Reasons for these variations were usually ascribed to differences in germplasms and environmental/climate factors of the cultivation [12], or were blamed to the sensitivity of CaDs and TNs to light and temperature [13]. The great variation in qualities of Danshen would certainly affect the qualities and clinical efficacies of its products and derived preparations, and therefore has become one of the most important problems pharmacognostic researches tried to overcome.

Drying is the most common and elementary procedure in the post-harvest process which affects the quality and value of medicinal plants [14,15]. The general belief is that levels of bioactive components in medicinal plants were a pre-harvest accumulation and were decreased in the post-harvest drying process along as the temperature increased and the duration was prolonged [16,17]. Therefore, the fundamental target of research on drying processes for medicinal plants up to now was how to best retain the initial levels of bioactive ingredients, and hence the freeze-drying protocol was recommended as the most suitable method [18]. However, from the view of plant physiology, the newly harvested fresh plant materials, especially roots, are still physiologically active organs and the drying process is a *bona fide* dehydration stress to these organs. Thus, the post-harvest drying process, especially at its early stage, could induce a series of anti-dehydration mechanisms including the production or increase of related secondary metabolites of these organs [19,20]. That is to say, pharmacologically important ingredients of medicinal plants might emerge or increase during a certain period of drying in post-harvest processing. The drying-induced increase of bioactive components might especially be true for some root materials and some types of secondary metabolites with important ecological functions [19,20]. This physiological peculiarity of the post-harvest plant materials has not be documented aside from the mention of its role in preventing water loss in the drying of fresh ginseng [21].

To test this hypothesis, we carried out a series of exploratory works on the post-harvest TCM drying process. Here, we displayed one of the important findings on roots of *S. miltiorrhiza.* Like most other TCMs, the universally applied way to dry this herb is through a sun-curing process. Usually, this process will be prolonged for at least one month to reach the standard level of moisture (≤13%) as documented in the Chinese Pharmacopoeia [10]. The changing trends of bioactive components during this process have not been reported hitherto, nor have researches on the ideal method for drying this herb. In the present paper, we report that SaB, the most abundant and important active ingredient of Danshen, was unexpectedly a product of the post-harvest drying process. The values of major tanshinones were also obviously increased during the drying process period. The results were of great value for promoting and stabilizing the quality of Danshen, and were also helpful for guiding similar investigations on other TCMs. 

## 2. Results and Discussion

Two independent experiments were conducted by Li in 2007–2009 (T1) and Wang in 2009–2010 (T2), respectively. In T1, the drying temperature was set in the range of 50~120 °C. Results showed that the value of SaB was still increasing until the temperature of 120 °C. Therefore, we designed T2 in the range of 90~160 °C for determining the inflection point of SaB. Although there were minor discrepancies in some values of the two experiments due to differences of initial materials and operation, trends of the variations were similar and results of the experiments were mutually verified. 

### 2.1. Dehydration Curves

**Figure 2:**
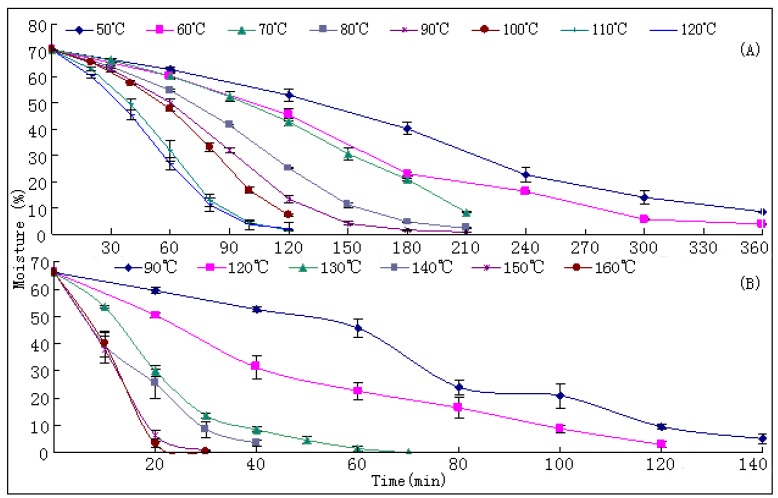
The drying curves of samples at different temperature. (**A**) results of experiment 1; (**B**) results of experiment 2.

The dehydration curves of fresh samples at different drying temperatures are illustrated in Figure 2. The results revealed that the drying efficacies were significantly temperature dependent. Durations required to reach the standard moisture (≤13%) [10] from their initial values (~70%) of fresh samples were significantly shortened as the drying temperature increased (Table 1). The drying duration at 120 °C was 4.5 times faster than that at 50 °C (T1). A significant positive correlation (p < 0.01) between drying rates and drying temperatures was observed (Table 1). 

Traditionally, the newly harvested Danshen roots were dried in the sun and this process should last about one month. The only criterion for the products was the level of moisture. Such a long duration of drying in the sun should certainly result in a severe change of bioactive components of the materials. To reasonably design proper drying protocols for Danshen is an urgent task. Our results provide useful information for this purpose.

**Table 1:** Drying efficacies and levels of five analytes in dried samples with standard moisture (S-values) *^a^*.

T *^b^*(°C)	Time (min)	Moisture (%)	Contents of analytes (mg/g, dry weight)					
			SaB	dTNI	cTN	TNI	TNIIa	TTN
50	360	8.55 ± 0.22	2.02 ± 0.20	0.14 ± 0.01	0.88 ± 0.06	0.98 ± 0.13	2.49 ± 0.28	4.49 ± 0.11
60	300	5.69 ± 0.11	10.99 ± 0.20	0.12 ± 0.01	0.82 ± 0.03	0.88 ± 0.04	2.31 ± 0.06	4.13 ± 0.01
70	210	8.26 ± 0.21	16.85 ± 0.56	0.15 ± 0.01	0.76 ± 0.08	1.05 ± 0.07	2.19 ± 0.14	4.15 ± 0.11
80	150	11.18 ± 0.97	23.10 ± 0.60	0.13 ± 0.01	0.73 ± 0.01	0.82 ± 0.03	2.11 ± 0.06	3.79 ± 0.03
90	150	4.08 ± 0.69	28.36 ± 1.09	0.16 ± 0.01	0.94 ± 0.01	1.16 ± 0.05	2.68 ± 0.14	4.94 ± 0.13
100	120	7.16 ± 0.41	27.66 ± 1.53	0.15 ± 0.02	0.90 ± 0.08	1.00 ± 0.12	2.47 ± 0.27	4.52 ± 0.14
110	80	12.92 ± 2.46	36.21 ± 1.46	0.18 ± 0.00	1.16 ± 0.03	1.58 ± 0.05	3.66 ± 0.08	6.58 ± 0.05
120	80	11.32 ± 0.25	40.0 ± 0.78	0.22 ± 0.01	1.23 ± 0.02	1.83 ± 0.02	3.62 ± 0.09	6.90 ± 0.13
90	120	9.57 ± 0.84	18.82 ± 0.48	0.21 ± 0.01	1.38 ± 0.06	0.17 ± 0.02	1.11 ± 0.06	2.87 ± 0.15
120	100	8.87 ± 1.36	33.78 ± 0.67	0.30 ± 0.01	3.11 ± 0.06	0.62 ± 0.03	2.99 ± 0.06	7.02 ± 0.16
130	40	8.50 ± 0.85	39.60 ± 2.81	0.35 ± 0.01	2.62 ± 0.02	2.24 ± 0.09	3.42 ± 0.09	8.63 ± 0.20
140	30	8.57 ± 2.98	33.12 ± 2.94	0.38 ± 0.02	2.12 ± 0.12	2.02 ± 0.12	3.06 ± 0.11	7.58 ± 0.37
150	20	6.47 ± 1.53	34.14 ± 1.54	0.23 ± 0.02	1.53 ± 0.21	1.17 ± 0.13	1.42 ± 0.14	4.35 ± 0.50
160	20	3.07 ± 1.03	32.46 ± 1.78	0.34 ± 0.01	2.22 ± 0.06	0.68 ± 0.02	1.63 ± 0.06	4.87 ± 0.15

*^a^* Results were mean values of triplicate assays; The upper and lower potions were results of two independent experiments; *^b^* Temperature.

### 2.2. Determination of Analytes

**Figure 3:**
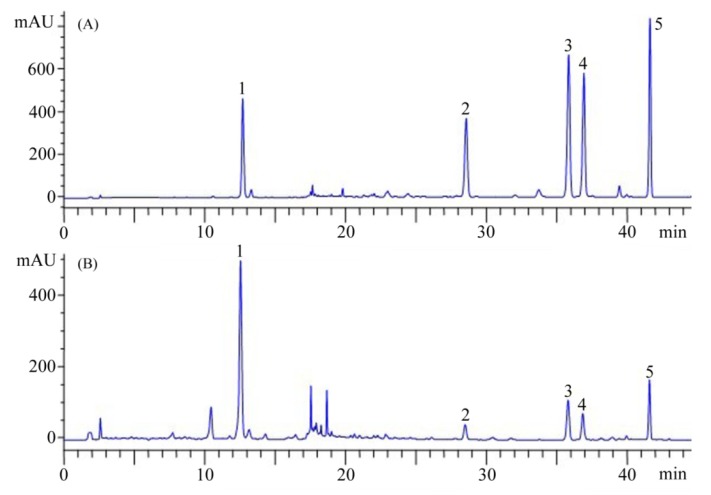
Typical chromatograms of standard mixture solution (**A**) and sample (**B**). (1) salvianolic acid B (SaB); (2) dihydrotanshinone I (dTN); (3) cryptotanshinone (cTN); (4) tanshinone I (TNI); (5) tanshinone IIA (TNIIa).

Details on HPLC method development and validation for simultaneous determination of the major five analytes in Danshen have been described in our former paper [22]. Briefly, the HPLC method employed resulted in a suitable resolution for their simultaneous determination (Figure 3). 

The regression equation and test range (in brackets) for each analyte was: *Y* = 24.50*X* − 46.00 (2.66–850 µg/mL) (**1**); *Y* = 67.27*X* + 11.43 (0.66–210 µg/mL) (**2**); *Y* = 43.18*X* + 11.9 (1.63–520 µg/mL) (**3**); *Y* = 84.66*X* + 62.51 (1.00–320 µg/mL) (**4**) and *Y* = 96.50*X* − 76.02 (0.81–260 µg/mL) (**5**). All calibration curves showed good linear regression (*r*^2^ > 0.9999) within test ranges. The limit of detection (LOD) and quantification (LOQ) for each standard compound were: 0.14 and 0.34 µg/mL (**1**); 0.03 and 0.11 µg/mL (**2**); 0.08 and 0.20 µg/mL (**3**); 0.04 and 0.13 µg/mL (**4**); 0.02 and 0.06 µg/mL (**5**). The recoveries of the five investigated components were within the range 97.9–105.3%. The relative standard deviations *(RSD*) of intra- and inter-day variability at different concentrations were in the range 0.27–2.10% and 1.44–3.94%, respectively. The employed HPLC approach in this study was accurate and precious for content determination of major five ingredients in samples [22]. 

Detailed results of determination on samples from suitable sampling points in process of drying at different temperatures were attached in Appendix A-B. Contents of five analytes in samples with standard moisture (S-values) and their apical values (A-values) at different drying temperature were summarized in Table 1 and Table 2, respectively.

**Table 2:** The apical values of five analytes in Danshen (A-values) and their corresponding moisture and drying duration *^a^*.

		SaB	dTN	cTN	TN I	TN II_A_	TTNs
50 °C	Contents *^b^*	4.77 ± 0.17	0.23 ± 0.02	1.21 ± 0.08	1.57 ± 0.21	4.04 ± 0.05	6.93 ± 0.08
	Duration *^c^*	300	60	120	120	60	60
	Moisture *^d^*	13.99 ± 2.71	62.61 ± 1.16	52.88 ± 2.32	52.88 ± 2.32	62.61 ± 1.16	62.61 ± 1.16
60 °C	Contents *^b^*	10.99 ± 0.20	0.20 ± 0.00	1.27 ± 0.04	1.26 ± 0.06	4.01 ± 0.12	7.05 ± 0.01
	Duration *^c^*	300	60	60	180	60	60
	Moisture *^d^*	5.69 ± 0.11	60.15 ± 0.95	60.15 ± 0.95	23.01 ± 0.79	60.15 ± 0.95	60.15 ± 0.95
70 °C	Contents *^b^*	18.01 ± 0.81	0.21 ± 0.01	1.45 ± 0.04	2.42 ± 0.05	4.63 ± 0.39	8.50 ± 0.10
	Duration *^c^*	180	90	120	90	90	90
	Moisture *^d^*	20.86 ± 0.48	52.38 ± 1.97	42.78 ± 1.36	52.38 ± 1.97	52.38 ± 1.97	52.38 ± 1.97
80 °C	Contents *^b^*	23.10 ± 0.60	0.23 ± 0.01	1.31 ± 0.05	1.58 ± 0.10	3.89 ± 0.17	7.01 ± 0.08
	Duration *^c^*	150	90	90	90	90	90
	Moisture *^d^*	11.18 ± 0.97	41.42 ± 0.47	41.42 ± 0.47	41.42 ± 0.47	41.42 ± 0.47	41.42 ± 0.47
90 °C	Contents *^b^*	32.60 ± 0.67	0.23 ± 0.01	1.46 ± 0.06	1.73 ± 0.06	4.41 ± 0.44	7.81 ± 0.10
	Duration *^c^*	120	90	90	60	60	90
	Moisture *^d^*	13.38 ± 1.52	31.87 ± 0.94	31.87 ± 0.94	31.87 ± 0.94	50.04 ± 1.29	31.87 ± 0.94
	Contents *^b^*	36.03 ± 2.72	0.30 ± 0.01	2.16 ± 0.05	2.42 ± 0.13	6.23 ± 0.33	11.11 ± 0.11
100 °C	Duration *^c^*	80	60	60	60	60	60
	Moisture *^d^*	33.15 ± 1.55	47.63 ± 1.03	47.63 ± 1.03	47.63 ± 1.03	47.63 ± 1.03	47.63 ± 1.03
110 °C	Contents *^b^*	38.10 ± 1.34	0.24 ± 0.01	1.62 ± 0.03	1.98 ± 0.06	5.04 ± 0.10	8.88 ± 0.05
	Duration *^c^*	60	60	60	60	60	60
	Moisture *^d^*	31.81 ± 4.03	31.81 ± 4.03	31.81 ± 4.03	31.81 ± 4.03	31.81 ± 4.03	31.81 ± 4.03
	Contents *^b^*	40.00 ± 0.78	0.25 ± 0.00	1.56 ± 0.03	1.91 ± 0.08	4.84 ± 0.19	8.56 ± 0.06
120 °C	Duration *^c^*	80	40	40	40	40	40
	Moisture *^d^*	11.32 ± 0.25	45.36 ± 1.90	45.36 ± 1.90	45.36 ± 1.90	45.36 ± 1.90	45.36 ± 1.90
90 °C	Contents *^b^*	18.82 ± 0.48	0.21 ± 0.01	1.58 ± 0.11	0.90 ± 0.18	1.11 ± 0.06	3.17 ± 0.35
	Duration *^c^*	120	120	60	0	120	40
	Moisture ***^d^***	9.57 ± 0.84	9.57 ± 0.84	45.70 ± 3.37	66.10 ± 0.14	9.57 ± 0.84	52.53 ± 0.91
120 °C	Contents *^b^*	35.42 ± 1.14	0.40 ± 0.04	3.23 ± 0.43	0.90 ± 0.18	2.99 ± 0.06	7.02 ± 0.16
	Duration *^c^*	80	80	80	0	100	100
	Moisture ***^d^***	16.43 ± 3.71	16.43 ± 3.71	16.43 ± 3.71	66.10 ± 0.14	8.87 ± 1.36	8.87 ± 1.36
130 °C	Contents *^b^*	39.60 ± 2.81	0.54 ± 0.04	2.82 ± 0.25	3.48 ± 0.12	3.61 ± 0.09	10.45 ± 0.50
	Duration *^c^*	40	60	60	60	60	60
	Moisture ***^d^***	8.50 ± 0.85	1.57 ± 0.84	1.57 ± 0.84	1.57 ± 0.84	1.57 ± 0.84	1.57 ± 0.84
140 °C	Contents *^b^*	38.84 ± 2.23	0.41 ± 0.02	2.71 ± 0.11	2.87 ± 0.19	3.83 ± 0.20	9.17 ± 0.41
	Duration *^c^*	20	40	20	40	20	20
	Moisture ***^d^***	25.33 ± 5.48	3.57 ± 1.02	25.33 ± 5.48	3.57 ± 1.02	25.33 ± 5.48	25.33 ± 5.48
150 °C	Contents *^b^*	34.14 ± 1.54	0.32 ± 0.03	2.41 ± 0.28	1.52 ± 0.32	2.38 ± 0.25	6.62 ± 0.88
	Duration *^c^*	20	30	10	10	10	10
	Moisture ***^d^***	6.47 ± 1.53	0.94 ± 0.17	37.80 ± 5.02	37.80 ± 5.02	37.80 ± 5.02	37.80 ± 5.02
160 °C	Contents *^b^*	32.46 ± 1.78	0.50 ± 0.04	2.51 ± 0.09	2.52 ± 0.07	2.41 ± 0.46	6.68 ± 0.73
	Duration *^c^*	20	30	10	30	10	10
	Moisture ***^d^***	3.07 ± 1.03	0.20 ± 0.133	39.87 ± 4.733	0.20 ± 0.13	39.87 ± 4.73	39.87 ± 4.73

*^a^* Results are mean values of triplicate assays; Content was the value of dry weight; The upper and lower potions were results of two independent experiments. *^b^* units: mg/g; ^c^ units: min; *^d^* units: %.

### 2.3. Production of SaB

**Figure 4:**
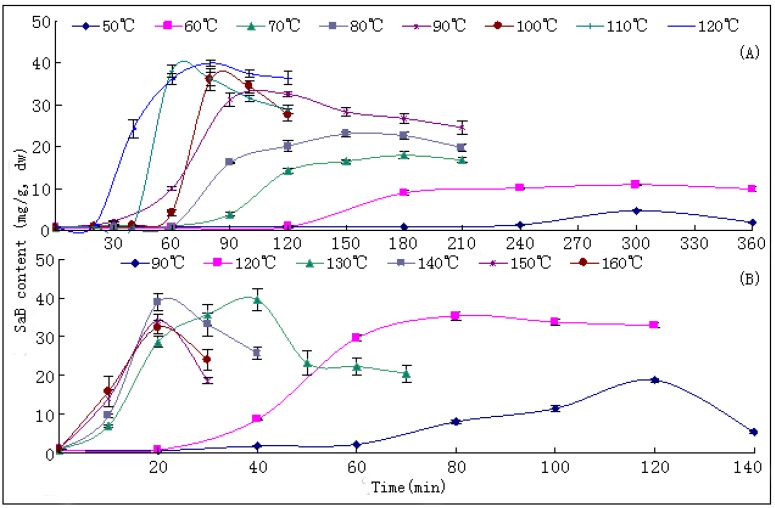
Production and variation curves of salvianolic acid B in Danshen during the drying process at different temperature. (**A**) results of experiment 1; (**B**) results of experiment 2.

**Figure 5:**
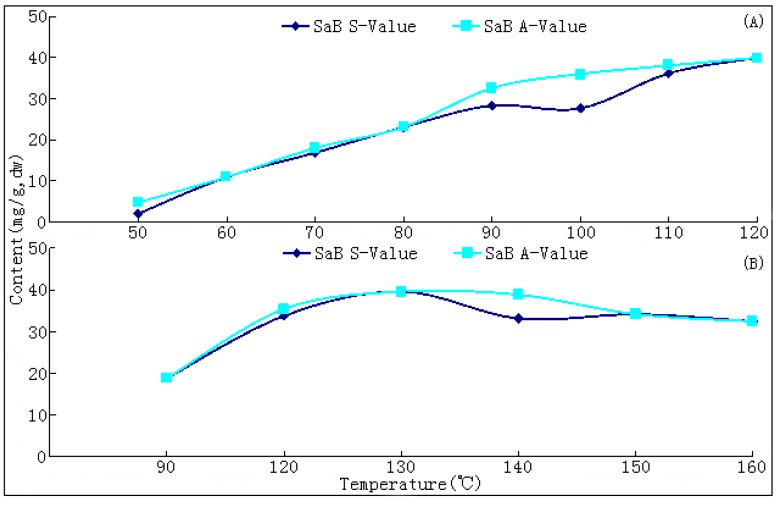
Contents of salvianolic acid B in sufficiently dried samples (S-values) and their apical values (A-values) at different drying temperature. (**A**) results of experiment 1; (**B**) results of experiment 2.

The most marked and unexpected result of our research was the finding of SaB, the most important and abundant bioactive component of Danshen, being a product of the post-harvest drying process. In both tests, only trace amounts (<1.0 mg/g of dry weight, dw) of SaB were found in fresh root materials; while a surge of SaB content was observed during the drying process at all temperatures, especially when these were above 70 °C (Figure 4). Its maximal value of around 40.0 mg/g dw was reached in drying at 120 (T1) and 130 °C (T2) (Table 1; Table 2; Figure 4 and Figure 5). Higher temperatures tended to result a higher yield of SaB and a shorter duration to reach this value. The S-values of SaB and their accelerating rates increased distinctly along with the elevation of drying temperature in the range of 50~130 °C and showed a significant positive correlation (p < 0.01) (Table 1). Additionally, an A-value of SaB at each drying temperature was observed usually ahead of the appearance of S-values in a relatively shorter drying time and higher sample moisture (Figure 5). All variations of SaB contents showed reverse V-shaped curves and the production of SaB showed a significant negative correlation (p < 0.01) with moistures in the up-curve. After the A-value levels of SaB decreased distinctly long with the prolonging of drying process and the degeneraion rates also showed a significant positive correlation with temperature (*p* < 0.05).

SaB has been revealed to be the most abundant ingredient of Danshen in most research publications up to now [7,9,11]. Our result demonstrated for the first time that this compound was actually not an essential ingredient accumulated in the growing and pre-harvest stage in roots of *S. miltiorrhiza*, but instead was a post-harvest product of the drying process. To our knowledge, such a remarkable promoting effect of the drying process on levels of bioactive components is also the first report in the field research on post-harvest treatment of medicinal plants. The absence of this knowledge in the literature might be ascribed to the reason that former investigations were all focused on the quality analysis on sufficiently dried herbs and neglected the analysis on fresh materials. A significant negative correlation between production of SaB and levels of moisture meant that this compound might be an important anti-dehydration ingredient of the plant; and the mechanism might be closely related to its effective abilities in scavenging the oxygen free radicals (OFRs) [23]. However, the increase of SaB prolonged until 130 °C of drying was really a quite puzzling question. Upon careful examination we found that the actual temperature in the oven was around 90~100 °C within the first 1 h of drying at 130 °C. This fact could provide a partial explanation for our observation. The key precursors, intermediates and mechanisms for producing SaB during the drying process are now under exploration in our laboratory.

### 2.4. Increases and Variations of Tanshinones

**Figure 6:**
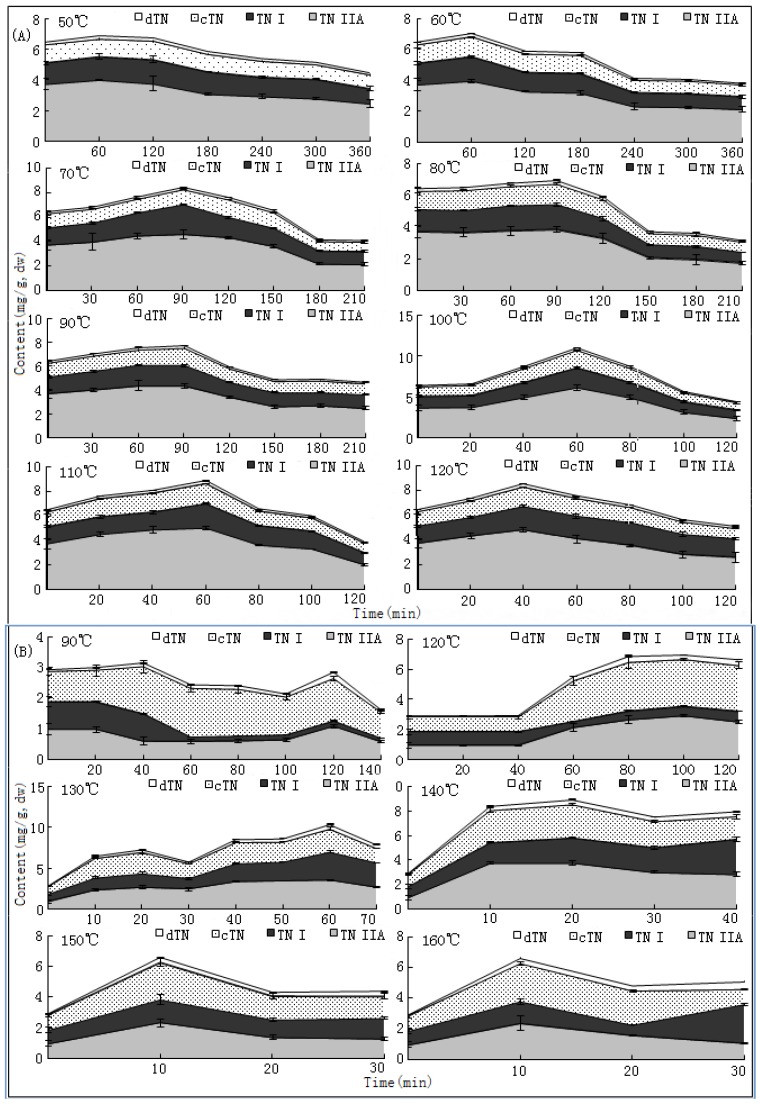
Increases and variation curves of tanshinones during the drying process at different temperature. (**A**) results of experiment 1; (**B**) results of experiment 2; Abbreviations: dTN (dihydrotanshinone I); cTN (cryptotanshinone); TNI (tanshinone I); TNIIa (tanshinone IIA).

Increases and variations of contents of dTNI, cTN, TNI and TNIIa in samples during the drying processes at different temperature are displayed in Figure 6. The results revealed that similar reserve V-shaped curves were observed for all values of tanshinones and their total amount (TTNs). Differently, TNs were initially the most abundant ingredients of fresh materials and obviously increased during the drying process. 

**Figure 7:**
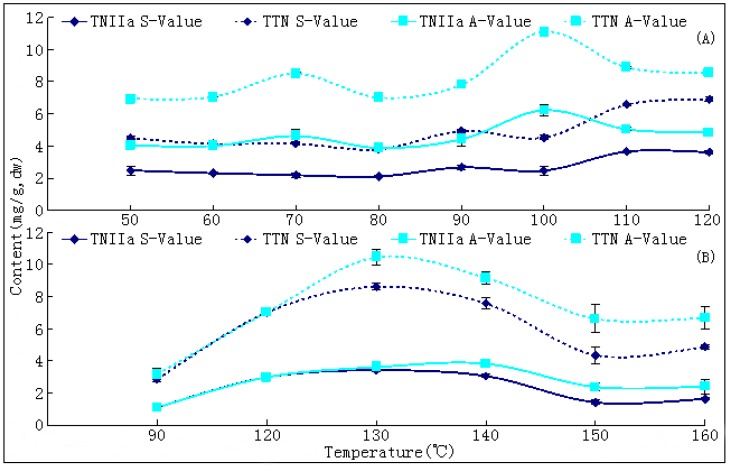
Contents of tanshinones in sufficiently dried samples (S-values) and their apical values (A-values) at different drying temperature. (**A**) results of experiment 1; (**B**) results of experiment 2; Abbreviations: TNIIa (tanshinone IIA); TTN (total tanshinones).

The A-values of TNIIa and TTNs were observed as 0.6% and 1.1% at 110 °C (T1) and as 0.38% and 1.05% at 130~140 °C (T2), respectively, and increased 50~60% and ~3.5 times from their initial values (Table 3, Figure 7). However, their S-values were decreased to 0.36% (TNIIa)/0.69% (TTNs) in T1, and 0.34% (TNIIa)/0.86% (TTNs) in T2, respectively (Table 2, Figure 7). The increase rates of each compound in the up-curves also showed significant positive correlations (*p* < 0.05) with temperatures and significant negative correlations (*p* < 0.05) with moistures. The S-values of each ingredient were obviously lower than their A-values due to materials of the former underwent a longer drying process. In addition, durations to reach A-values of each compound were obviously ahead of the duration required to obtain the A-value of SaB. These facts meant that TNs were much sensitive to temperature and drying duration than SaB.

Most existing literatures indicated that increasing temperature and longer duration of drying during post-harvest processing have an adverse effect on concentrations of bioactive constituents of medicinal plants [14,16,17,18]. Only a few reports demonstrated that an increased temperature within a proper range could have a positive effect on the quality of these materials [24,25]. Our results revealed that significant increases of TNs levels together with the induced production of SaB in roots of *S. miltiorrhiza* appeared in the early stage of the post-harvest drying process. The reason for these results could be explained by the fact that both SaB and TNs were effective scavengers of OFRs which must play important functions for the plant against dehydration stress. At the early stage of post-harvest drying, the roots were still physiologically active and thus might induce production or increases in these compounds in order to protect against the fatal injury of an OFRs burst under such stress [19,20,23]. Our speculation and experimental results might overturn the general recognition that the believed values of bioactive components of medicinal plants were accumulated during the growth period of the pre-harvest and were decreased in the post-harvest processing. The drying-induced increase of bioactive components might be true for some roots materials and some types of secondary metabolites with important ecological functions. This new notion could help significantly promote rather than simply retaining the qualities of Danshen and some other similar medicinal plants. It is an interesting subject in the field of production and quality control of medicinal plants and needs to be further investigated.

## 3. Experimental

### 3.1. Plant Materials

Experiments in this paper were separately conducted by two graduate students, Mr. Xiao-Bing Li (T1) and Mr. Wei Wang (T2), during 2007~2010. Fresh root samples of *S. miltiorrhiza* (each about 20 Kg) were collected from Linqu County, Shandong Province of China, during the harvest season in October 1~15 of 2007 and 2009. The fresh roots were taken to the laboratory within two days and immediately sliced in 2~3 mm pieces. The sliced materials were mixed well and stored at −20 °C in a refrigerator until assay. The samples were authenticated by Dr. Tong-Shui Zhou and the voucher specimens were deposited in the Herbarium of Fudan University, Shanghai, China.

### 3.2. Drying Process and Temperature Design

The drying process of the frozen sliced materials was conducted in a thermostatic oven. In T1, eight groups of samples were dried at temperatured from 50~120 °C in 10 °C intervals, respectively. Accurately weighted materials (30.0 g) were evenly placed in a ф 9.0 cm watch-glass. The oven was preheated to the set temperature, put into the materials and the experimental duration was started. The amount of materials and watch-glasses for each test group were carefully designed according to the sampling interval and frequency at each drying temperature. For each sampling point, two batches each in three watch-glasses were randomly extracted from the oven during the drying process. The sampled watch-glasses were placed in a desiccator for cooling and then, the contained samples were pulverized separately (80 mesh) and weighed accurately. These samples were used for the determination of moisture and contents of analytes, respectively. The sampling interval and frequency at each drying temperature were determined according to the difference of drying efficiency at each temperature, *i.e.*,1 h intervals in total 6 h for 50 and 60 °C; 30 min intervals in total 3.5 h for 70, 80 and 90 °C; and 20 min intervals in total 2 h for 100, 110 and 120 °C. In T2, six further groups of tests from 90~160 °C were designed to determinate the inflection point of SaB change. The drying and sampling procedures were consistent with T1. 

### 3.3. Determination of Moisture

Moisture of samples from different sampling points was determined in triplicate by Method I as described in the 2010 Chinese Pharmacopoeia [10]. Accurately weighed powdered samples were dried at 105 °C in an oven at least for 5 h until a constant weight was obtained. After cooling in a desiccator, they were accurately weighed and the loss of water calculated. 

### 3.4. Determination of Analytes

The contents of five major bioactive components in Danshen, namely SaB (**1**), dTN (**2**), cTN (**3**), TNI (**4**) and TNIIa (**5**), were determined by high performance liquid chromatography (HPLC). Details of the method have been published previously [22], and are briefly described below:

*Standard chemicals and* *reagents*. High purity (>98%) cTN, dTN, TNI and TNIIa (purified from the dried roots of *S. miltiorrhiza* in our laboratory) and SaB (purchased from Shanghai R&D Center for Standardization of TCMs, China) were used as standards for quantitative analysis. HPLC grade acetonitrile and trifluoroacetic acid (TFA) were products of Merck (Darmstadt, Germany). Triple distilled water produced using an 1810D water distiller (Shanghai Shenke Ltd., Shanghai, China) was used for all extraction and separation procedures. All other reagents were of analytical grade and obtained from local companies. 

*Apparatus and* *chromatographic* *conditions*. An Agilent Series 1100 LC instrument (Agilent Technologies, Waldbronn, Germany) equipped with an on-line degasser, a quaternary pump, a diode-array detector (DAD) and a 20 µL sample loop manual injector was used for sample analysis. The equipment was automatically controlled by ChemStation (Rev.A. 07.01) software. The column configuration was an YMC-Pack Pro C18 fast analysis column (3 µm, 4.6 mm × 150 mm) connected to an Industries C_18_ guard column (5 µm, 4.0 mm × 20 mm). The mobile phase consisted of solvent A (acetonitrile) and B (0.1% aqueous trifluoroacetic acid (aTFA), *v/v*). A gradient elution program was used as follows: 20–27% A (*v/v*) at 0–14 min, 27–47% A (*v/v*) at 14–15 min, 47–52% A (*v/v*) at 15–31 min and 52–85% A (*v/v*) at 31–44 min. The flow rate was 1.0 mL/min and the injection volume was 20 µL. Re-equilibration duration was 10 min between individual runs. The diode-array detector was set at 280 nm for SaB and 254 nm for tanshinones, respectively.

*Calibration and* *method* *validation*. Standard stock solutions of the five analytes were prepared by dissolving the accurately weighed dTN (2.1 mg), cTN (5.2 mg), TNI (3.2 mg), TNIIa (2.6 mg) and SaB (8.5 mg) in a 10 mL volumetric flask with 70% aqueous methanol (aM). The solution was then diluted with 70% aM to appropriate concentrations for the assessment of linearity and method validation. Seven concentration levels were prepared for calibration and linear analysis of each standard compound. Peak area (*Y*) and concentration (*X*) for each compound were analyzed to calculate the calibration curve and correlation coefficient (*r*). The LODs and LOQs under the present chromatographic conditions were determined at a signal-to-noise (*S/N*) ratio of 3 and 10, respectively. The recovery tests were assessed with an appropriate amount of herb sample spiked with three different quantities (low, medium and high) of authentic standards. The intra-day variability was examined five times within one day, and the inter-day precision was calculated from nine determinations over three days (three determinations per day) for low, medium and high concentrations of authentic standard solutions. All standard solutions of various concentrations were stored at 4 °C in a refrigerator until assayed. Each test was analyzed in triplicate.

*Sample* *assay*. Accurately weighed (~0.50 g) powders (80 mesh) of samples were extracted twice with 70% aM (20 mL) in an ultrasonic bath at room temperature for 20 min. The supernatants after centrifugation (3000 rpm for 10 min) were combined and diluted with 70% aM to 50 mL. The solutions were filtered through a 0.45 µm nylon syringe filter (Millex-HN, Massachusetts, USA) before HPLC analysis. Triplicate samples for each sampling point were analyzed. 

### 3.5. Statistic Analysis

All data were the mean values of three independent experiments. The statistic analysis, *i.e.*, the least significance difference and the bivariate correlation, was conducted using the SPSS 11.0 program. 

## 4. Conclusions

In this paper, we revealed a surge of SaB levels and significant increases of TNs in roots of *S. miltiorrhiza* in the early stages of post-harvest drying at different oven temperatures. Variations of all analytes showed reserve V-shaped curves during the entire drying process. The increases of each compound in the up-curve showed significant positive correlations with drying temperature and significant negative correlations with sample moistures. The reason for these results was ascribed to the fact that both SaB and TNs were effective scavengers of OFRs which must play important functions for the protection of plants against dehydration stress. This hypothesis is interesting and useful for the production and quality control of medicinal plants and needs to be further investigated. The present results were of great value for uncovering the mysterious nature of the great quality variation of Danshen and for providing a reasonable strategy to overcome this problem. 

## Appendix: Contents of moisture and analytes in Danshen during drying at 50~120 °C (Appendix A) and 90~160 °C (Appendix B).

**Appendix A-1:** Contents of moisture and analytes in Danshen during drying at 50 and 60 °C (n = 3).

Temp.	Analytes	Drying time (min)						
		0	60	120	180	240	300	360
50 °C	Moisture (%)	70.10 ± 0.15	62.61 ± 1.16	52.88 ± 2.32	40.13 ± 2.35	22.58 ± 2.72	13.99 ± 2.71	8.55 ± 0.22
	Sa B (mg/g)	0.79 ± 0.03	0.80 ± 0.04	0.86 ± 0.01	0.90 ± 0.02	1.39 ± 0.06	4.77 ± 0.17	2.02 ± 0.20
	dTN (mg/g)	0.18 ± 0.02	0.23 ± 0.02	0.22 ± 0.01	0.20 ± 0.00	0.18 ± 0.01	0.17 ± 0.00	0.14 ± 0.01
	cTN (mg/g)	1.17 ± 0.15	1.14 ± 0.04	1.21 ± 0.08	1.13 ± 0.02	1.03 ± 0.04	0.94 ± 0.02	0.88 ± 0.06
	TN I (mg/g)	1.39 ± 0.11	1.52 ± 0.18	1.57 ± 0.21	1.45 ± 0.01	1.25 ± 0.05	1.26 ± 0.04	0.98 ± 0.13
	TN II_A_ (mg/g)	3.77 ± 0.38	4.04 ± 0.05	3.79 ± 0.47	3.12 ± 0.07	2.97 ± 0.13	2.82 ± 0.04	2.49 ± 0.28
	TTNs (mg/g)	6.51 ± 0.22	6.93 ± 0.08	6.79 ± 0.17	5.90 ± 0.04	5.43 ± 0.06	5.19 ± 0.04	4.49 ± 0.11
60°C	Moisture (%)	70.10 ± 0.15	60.15 ± 0.95	45.36 ± 2.21	23.01 ± 0.79	16.30 ± 0.43	5.69 ± 0.11	3.87 ± 0.17
	Sa B (mg/g)	0.79 ± 0.03	0.64 ± 0.05	0.90 ± 0.28	8.99 ± 0.56	10.13 ± 0.11	10.99 ± 0.20	9.95 ± 0.56
	dTN (mg/g)	0.18 ± 0.02	0.20 ± 0.00	0.19 ± 0.00	0.18 ± 0.01	0.14 ± 0.01	0.12 ± 0.01	0.11 ± 0.01
	cTN (mg/g)	1.17 ± 0.15	1.27 ± 0.04	1.18 ± 0.02	1.16 ± 0.04	0.80 ± 0.02	0.82 ± 0.03	0.76 ± 0.06
	TN I (mg/g)	1.39 ± 0.11	1.57 ± 0.08	1.24 ± 0.04	1.26 ± 0.06	0.90 ± 0.03	0.88 ± 0.04	0.83 ± 0.09
	TN II_A_ (mg/g)	3.77 ± 0.38	4.01 ± 0.12	3.32 ± 0.03	3.24 ± 0.14	2.36 ± 0.21	2.31 ± 0.06	2.17 ± 0.17
	TTNs (mg/g)	6.51 ± 0.04	7.05 ± 0.01	5.93 ± 0.00	5.84 ± 0.01	4.20 ± 0.02	4.13 ± 0.01	3.87 ± 0.02

**Appendix A-2:** Contents of moisture and analytes in Danshen during drying at 70, 80 and 90 °C (n = 3).

Temp.	Analytes	Drying time (min)							
		0	30	60	90	120	150	180	210
70 °C	Moisture (%)	70.10 ± 0.15	66.11 ± 0.65	60.17 ± 1.09	52.38 ± 1.97	42.78 ± 1.36	30.60 ± 2.24	20.86 ± 0.48	8.26 ± 0.21
	Sa B (mg/g)	0.79 ± 0.03	1.38 ± 0.12	1.11 ± 0.02	3.9 ± 0.40	14.3 ± 0.57	16.56 ± 0.44	18.01 ± 0.81	16.85 ± 0.56
	dTN (mg/g)	0.18 ± 0.02	0.18 ± 0.01	0.19 ± 0.01	0.21 ± 0.01	0.21 ± 0.01	0.20 ± 0.01	0.15 ± 0.01	0.15 ± 0.01
	cTN (mg/g)	1.17 ± 0.15	1.15 ± 0.01	1.14 ± 0.05	1.24 ± 0.05	1.45 ± 0.04	1.28 ± 0.05	0.79 ± 0.04	0.76 ± 0.08
	TN I (mg/g)	1.39 ± 0.11	1.54 ± 0.07	1.89 ± 0.07	2.42 ± 0.05	1.64 ± 0.07	1.47 ± 0.05	1.03 ± 0.03	1.05 ± 0.07
	TN II_A_(mg/g)	3.77 ± 0.38	4.01 ± 0.70	4.5 ± 0.21	4.63 ± 0.39	4.37 ± 0.11	3.65 ± 0.15	2.24 ± 0.06	2.19 ± 0.14
	TTNs (mg/g)	6.51 ± 0.22	6.88 ± 0.09	7.72 ± 0.08	8.50 ± 0.10	7.67 ± 0.06	6.60 ± 0.08	4.21 ± 0.05	4.15 ± 0.11
80 °C	Moisture (%)	70.10 ± 0.15	63.75 ± 0.58	54.72 ± 0.51	41.42 ± 0.47	25.12 ± 0.33	11.18 ± 0.97	4.71 ± 0.04	2.28 ± 0.17
	Sa B (mg/g)	0.79 ± 0.03	0.82 ± 0.04	0.94 ± 0.04	16.20 ± 0.19	20.2 ± 1.37	23.1 ± 0.60	22.7 ± 0.78	19.80 ± 0.73
	dTN (mg/g)	0.18 ± 0.02	0.20 ± 0.00	0.21 ± 0.00	0.23 ± 0.01	0.20 ± 0.01	0.13 ± 0.01	0.13 ± 0.02	0.11 ± 0.00
	cTN (mg/g)	1.17 ± 0.15	1.28 ± 0.02	1.24 ± 0.04	1.31 ± 0.05	1.21 ± 0.11	0.73 ± 0.01	0.74 ± 0.09	0.68 ± 0.03
	TN I (mg/g)	1.39 ± 0.11	1.39 ± 0.04	1.57 ± 0.03	1.58 ± 0.10	1.25 ± 0.13	0.82 ± 0.03	0.82 ± 0.08	0.66 ± 0.02
	TN II_A_ (mg/g)	3.77 ± 0.38	3.7 ± 0.28	3.82 ± 0.29	3.89 ± 0.17	3.34 ± 0.33	2.11 ± 0.06	2.00 ± 0.30	1.78 ± 0.10
	TTNs (mg/g)	6.51 ± 0.22	6.57 ± 0.05	6.84 ± 0.07	7.01 ± 0.08	6.00 ± 0.17	3.79 ± 0.03	3.69 ± 0.14	3.23 ± 0.05
90 °C	Moisture (%)	70.10 ± 0.15	62.78 ± 1.10	50.04 ± 1.29	31.87 ± 0.94	13.38 ± 1.52	4.08 ± 0.69	1.63 ± 0.23	0.82 ± 0.05
	Sa B (mg/g)	0.79 ± 0.03	2.28 ± 0.16	9.99 ± 0.43	31.3 ± 1.61	32.60 ± 0.67	28.36 ± 1.09	26.8 ± 1.21	24.63 ± 1.56
	dTN (mg/g)	0.18 ± 0.02	0.20 ± 0.01	0.20 ± 0.02	0.23 ± 0.01	0.16 ± 0.01	0.16 ± 0.01	0.15 ± 0.00	0.13 ± 0.01
	cTN (mg/g)	1.17 ± 0.15	1.28 ± 0.12	1.29 ± 0.08	1.46 ± 0.06	1.13 ± 0.02	0.94 ± 0.01	0.97 ± 0.04	0.92 ± 0.02
	TN I (mg/g)	1.39 ± 0.11	1.56 ± 0.09	1.73 ± 0.06	1.72 ± 0.10	1.24 ± 0.03	1.16 ± 0.05	1.10 ± 0.04	1.13 ± 0.05
	TN II_A_ (mg/g)	3.77 ± 0.38	4.08 ± 0.15	4.41 ± 0.44	4.40 ± 0.22	3.47 ± 0.10	2.68 ± 0.14	2.76 ± 0.14	2.54 ± 0.11
	TTNs (mg/g)	6.51 ± 0.22	7.12 ± 0.15	7.63 ± 0.15	7.81 ± 0.10	6.00 ± 0.04	4.94 ± 0.13	4.98 ± 0.06	4.72 ± 0.05

**Appendix A-3:** Contents of moisture and analytes in Danshen during drying at100, 110 and 120 °C (n = 3).

Temp.	Analytes	Drying time (min)						
		0	20	40	60	80	100	120
100 °C	Moisture (%)	70.10 ± 0.15	65.37 ± 0.14	57.56 ± 0.65	47.63 ± 1.03	33.15 ± 1.55	16.77 ± 1.15	7.16 ± 0.41
	Sa B (mg/g)	0.79 ± 0.03	0.96 ± 0.04	1.24 ± 0.11	4.35 ± 0.72	36.03 ± 2.72	34.34 ± 1.33	27.66 ± 1.53
	dTN (mg/g)	0.18 ± 0.02	0.20 ± 0.01	0.27 ± 0.02	0.30 ± 0.01	0.26 ± 0.00	0.18 ± 0.01	0.15 ± 0.02
	cTN (mg/g)	1.17 ± 0.15	1.25 ± 0.05	1.71 ± 0.10	2.16 ± 0.05	1.74 ± 0.02	1.1 ± 0.05	0.9 ± 0.08
	TN I (mg/g)	1.39 ± 0.11	1.44 ± 0.12	1.84 ± 0.14	2.42 ± 0.13	1.85 ± 0.09	1.28 ± 0.11	1.00 ± 0.12
	TN II_A_ (mg/g)	3.77 ± 0.38	3.81 ± 0.23	5.03 ± 0.32	6.23 ± 0.33	5.01 ± 0.26	3.20 ± 0.28	2.47 ± 0.27
	TTNs (mg/g)	6.51 ± 0.22	6.70 ± 0.08	8.85 ± 0.17	11.11 ± 0.11	8.86 ± 0.05	5.76 ± 0.10	4.52 ± 0.14
110 °C	Moisture (%)	70.10 ± 0.15	62.69 ± 0.65	49.42 ± 2.14	31.81 ± 4.03	12.92 ± 2.46	4.40 ± 0.42	1.47 ± 0.22
	Sa B (mg/g)	0.79 ± 0.03	0.84 ± 0.02	1.04 ± 0.16	38.10 ± 1.34	36.21 ± 1.46	31.62 ± 0.70	28.95 ± 1.01
	dTN (mg/g)	0.18 ± 0.02	0.20 ± 0.01	0.21 ± 0.02	0.24 ± 0.01	0.18 ± 0.00	0.17 ± 0.00	0.13 ± 0.00
	cTN (mg/g)	1.17 ± 0.15	1.45 ± 0.04	1.53 ± 0.01	1.62 ± 0.03	1.16 ± 0.03	1.05 ± 0.01	0.73 ± 0.02
	TN I (mg/g)	1.39 ± 0.11	1.44 ± 0.12	1.46 ± 0.14	1.98 ± 0.06	1.58 ± 0.05	1.44 ± 0.05	0.98 ± 0.06
	TN II_A_ (mg/g)	3.77 ± 0.38	4.53 ± 0.22	4.89 ± 0.31	5.04 ± 0.10	3.66 ± 0.08	3.36 ± 0.02	2.07 ± 0.07
	TTNs (mg/g)	6.51 ± 0.22	7.62 ± 0.08	8.09 ± 0.19	8.88 ± 0.05	6.58 ± 0.05	6.02 ± 0.02	3.91 ± 0.03
120 °C	Moisture (%)	70.10 ± 0.15	60.13 ± 0.66	45.36 ± 1.90	26.74 ± 2.37	11.32 ± 0.25	3.74 ± 0.19	2.12 ± 0.25
	Sa B (mg/g)	0.79 ± 0.03	1.34 ± 0.08	24.3 ± 2.15	36.2 ± 1.30	40.03 ± 0.78	37.5 ± 0.94	36.42 ± 1.59
	dTN (mg/g)	0.18 ± 0.02	0.20 ± 0.01	0.25 ± 0.00	0.21 ± 0.01	0.22 ± 0.01	0.20 ± 0.01	0.19 ± 0.01
	cTN (mg/g)	1.17 ± 0.15	1.33 ± 0.04	1.56 ± 0.03	1.43 ± 0.06	1.23 ± 0.02	1.01 ± 0.08	0.85 ± 0.03
	TN I (mg/g)	1.39 ± 0.11	1.51 ± 0.09	1.91 ± 0.08	1.85 ± 0.20	1.83 ± 0.02	1.62 ± 0.15	1.49 ± 0.11
	TN II_A_ (mg/g)	3.77 ± 0.38	4.35 ± 0.21	4.84 ± 0.19	4.13 ± 0.32	3.62 ± 0.09	2.88 ± 0.25	2.65 ± 0.38
	TTNs (mg/g)	6.51 ± 0.22	7.39 ± 0.08	8.56 ± 0.06	7.62 ± 0.12	6.90 ± 0.13	5.71 ± 0.13	5.18 ± 0.09

**Appendix B-1:** Contents of moisture and analytes in Danshen during drying at90 °C (n = 3).

Temp.	Analytes	Drying time (min)							
		0	20	40	60	80	100	120	140
90 °C	Moisture (%)	66.10 ± 0.14	59.47 ± 1.12	52.53 ± 0.91	45.70 ± 3.37	20.93 ± 4.50	24.03 ± 2.74	9.57 ± 0.84	5.13 ± 1.87
	Sa B (mg/g)	0.84 ± 0.09	0.61 ± 0.08	1.81 ± 0.10	2.22 ± 0.09	8.08 ± 0.51	11.47 ± 0.76	18.82 ± 0.48	5.43 ± 0.30
	dTN (mg/g)	0.06 ± 0.01	0.09 ± 0.01	0.12 ± 0.02	0.12 ± 0.01	0.12 ± 0.02	0.10 ± 0.010	0.21 ± 0.01	0.08 ± 0.02
	cTN (mg/g)	0.98 ± 0.12	1.02 ± 0.19	1.53 ± 0.19	1.58 ± 0.11	1.52 ± 0.11	1.23 ± 0.10	1.38 ± 0.06	0.84 ± 0.04
	TN I (mg/g)	0.90 ± 0.18	0.90 ± 0.03	0.90 ± 0.02	0.15 ± 0.01	0.16 ± 0.01	0.17 ± 0.01	0.17 ± 0.02	0.12 ± 0.01
	TN II_A_ (mg/g)	1.01 ± 0.18	1.01 ± 0.09	0.62 ± 0.12	0.61 ± 0.09	0.63 ± 0.05	0.66 ± 0.06	1.11 ± 0.06	0.60 ± 0.04
	TTNs (mg/g)	2.95 ± 0.49	3.02 ± 0.32	3.17 ± 0.35	2.46 ± 0.22	2.43 ± 0.18	2.16 ± 0.19	2.87 ± 0.15	1.64 ± 0.11

**Appendix B-2:** Contents of moisture and analytes in Danshen during drying at120 °C (n = 3).

Temp.	Analytes	Drying time (min)						
		0	20	40	60	80	100	120
120 °C	Moisture (%)	66.10 ± 0.14	50.43 ± 0.95	31.27 ± 4.38	22.67 ± 3.12	16.43 ± 3.71	8.87 ± 1.36	3.00 ± 1.18
	Sa B (mg/g)	0.84 ± 0.09	0.72 ± 0.02	8.6 ± 0.10	29.64 ± 0.77	35.42 ± 1.14	33.78 ± 0.67	32.91 ± 0.54
	dTN (mg/g)	0.06 ± 0.01	0.06 ± 0.00	0.09 ± 0.01	0.27 ± 0.01	0.40 ± 0.04	0.30 ± 0.01	0.40 ± 0.02
	cTN (mg/g)	0.98 ± 0.12	0.98 ± 0.01	0.98 ± 0.10	2.71 ± 0.29	3.23 ± 0.43	3.11 ± 0.06	3.03 ± 0.23
	TN I (mg/g)	0.90 ± 0.18	0.90 ± 0.01	0.90 ± 0.01	0.39 ± 0.02	0.58 ± 0.04	0.62 ± 0.03	0.71 ± 0.02
	TN II_A_ (mg/g)	1.01 ± 0.18	1.01 ± 0.01	1.01 ± 0.05	2.20 ± 0.25	2.72 ± 0.25	2.99 ± 0.06	2.57 ± 0.12
	TTNs (mg/g)	2.95 ± 0.49	2.95 ± 0.12	2.98 ± 0.17	5.57 ± 0.57	6.93 ± 0.77	7.02 ± 0.16	6.71 ± 0.39

**Appendix B-3:** Contents of moisture and analytes in Danshen during drying at130 and140 °C (n = 3).

Temp.	Analytes	Drying time (min)							
		0	10	20	30	40	50	60	70
130 °C	Moisture (%)	66.10 ± 0.14	53.43 ± 0.51	30.03 ± 1.97	13.63 ± 0.75	8.50 ± 0.85	4.57 ± 1.40	1.57 ± 0.84	0
	Sa B (mg/g)	0.84 ± 0.09	6.91 ± 0.32	28.62 ± 1.42	35.72 ± 2.61	39.60 ± 2.81	23.23 ± 3.02	22.34 ± 2.32	20.53 ± 2.21
	dTN (mg/g)	0.06 ± 0.01	0.27 ± 0.04	0.31 ± 0.03	0.21 ± 0.02	0.35 ± 0.01	0.41 ± 0.01	0.54 ± 0.04	0.43 ± 0.04
	cTN (mg/g)	0.98 ± 0.12	2.42 ± 0.08	2.64 ± 0.21	1.81 ± 0.14	2.62 ± 0.02	2.45 ± 0.03	2.82 ± 0.25	1.76 ± 0.03
	TN I (mg/g)	0.90 ± 0.18	1.51 ± 0.16	1.71 ± 0.15	1.32 ± 0.14	2.24 ± 0.09	2.34 ± 0.05	3.48 ± 0.12	3.04 ± 0.01
	TN II_A_ (mg/g)	1.01 ± 0.18	2.40 ± 0.15	2.72 ± 0.20	2.54 ± 0.19	3.42 ± 0.09	3.52 ± 0.05	3.61 ± 0.09	2.74 ± 0.09
	TTNs (mg/g)	2.95 ± 0.49	7.60 ± 0.43	8.38 ± 0.59	5.88 ± 0.49	8.63 ± 0.20	8.72 ± 0.13	10.45 ± 0.50	7.97 ± 0.17

**Appendix B-4:** Contents of moisture and analytes in Danshen during drying at 140 °C (n = 3).

Temp.	Analytes	Drying time (min)				
		0	10	20	30	40
140 °C	Moisture (%)	66.10 ± 0.14	39.50 ± 4.42	25.33 ± 5.48	8.57 ± 2.98	3.57 ± 1.02
	Sa B (mg/g)	0.84 ± 0.09	9.52 ± 0.42	38.84 ± 2.23	33.12 ± 2.94	25.72 ± 1.43
	dTN (mg/g)	0.06 ± 0.01	0.35 ± 0.05	0.39 ± 0.03	0.38 ± 0.02	0.41 ± 0.02
	cTN (mg/g)	0.98 ± 0.12	2.62 ± 0.18	2.71 ± 0.11	2.12 ± 0.12	1.83 ± 0.20
	TN I (mg/g)	0.90 ± 0.18	1.67 ± 0.04	2.04 ± 0.06	2.02 ± 0.12	2.87 ± 0.19
	TN II_A_ (mg/g)	1.01 ± 0.18	3.81 ± 0.06	3.83 ± 0.20	3.06 ± 0.11	2.90 ± 0.19
	TTNs (mg/g)	2.95 ± 0.49	8.45 ± 0.32	9.17 ± 0.41	7.58 ± 0.37	8.01 ± 0.60

**Appendix B-5:** Contents of moisture and analytes in Danshen during drying at 150 °C (n = 3).

Temp.	Analytes	Drying time (min)			
		0	10	20	30
150 °C	Moisture (%)	66.10 ± 0.14	37.80 ± 5.02	6.47 ± 1.53	0.94 ± 0.17
	Sa B (mg/g)	0.84 ± 0.09	14.12 ± 2.11	34.14 ± 1.54	18.65 ± 0.78
	dTN (mg/g)	0.06 ± 0.01	0.31 ± 0.03	0.23 ± 0.02	0.32 ± 0.03
	cTN (mg/g)	0.98 ± 0.12	2.41 ± 0.28	1.53 ± 0.21	1.42 ± 0.17
	TN I (mg/g)	0.90 ± 0.18	1.52 ± 0.32	1.17 ± 0.13	1.35 ± 0.08
	TN II_A_ (mg/g)	1.01 ± 0.18	2.38 ± 0.25	1.42 ± 0.14	1.33 ± 0.10
	TTNs (mg/g)	2.95 ± 0.49	6.62 ± 0.88	4.35 ± 0.50	4.42 ± 0.38

**Appendix B-6:** Contents of moisture and analytes in Danshen during drying at 160 °C (n = 3).

Temp.	Analytes	Drying time (min)			
		0	10	20	30
160 °C	Moisture (%)	66.10 ± 0.14	39.87 ± 4.73	3.07 ± 1.03	0.20 ± 0.13
	Sa B (mg/g)	0.84 ± 0.09	15.87 ± 3.82	32.46 ± 1.78	23.93 ± 2.74
	dTN (mg/g)	0.06 ± 0.01	0.34 ± 0.01	0.34 ± 0.01	0.50 ± 0.04
	cTN (mg/g)	0.98 ± 0.12	2.51 ± 0.09	2.22 ± 0.06	0.98 ± 0.03
	TN I (mg/g)	0.90 ± 0.18	1.42 ± 0.16	0.68 ± 0.02	2.52 ± 0.07
	TN II_A_ (mg/g)	1.01 ± 0.18	2.41 ± 0.46	1.63 ± 0.06	1.13 ± 0.03
	TTNs (mg/g)	2.95 ± 0.49	6.68 ± 0.73	4.87 ± 0.15	5.13 ± 0.17
